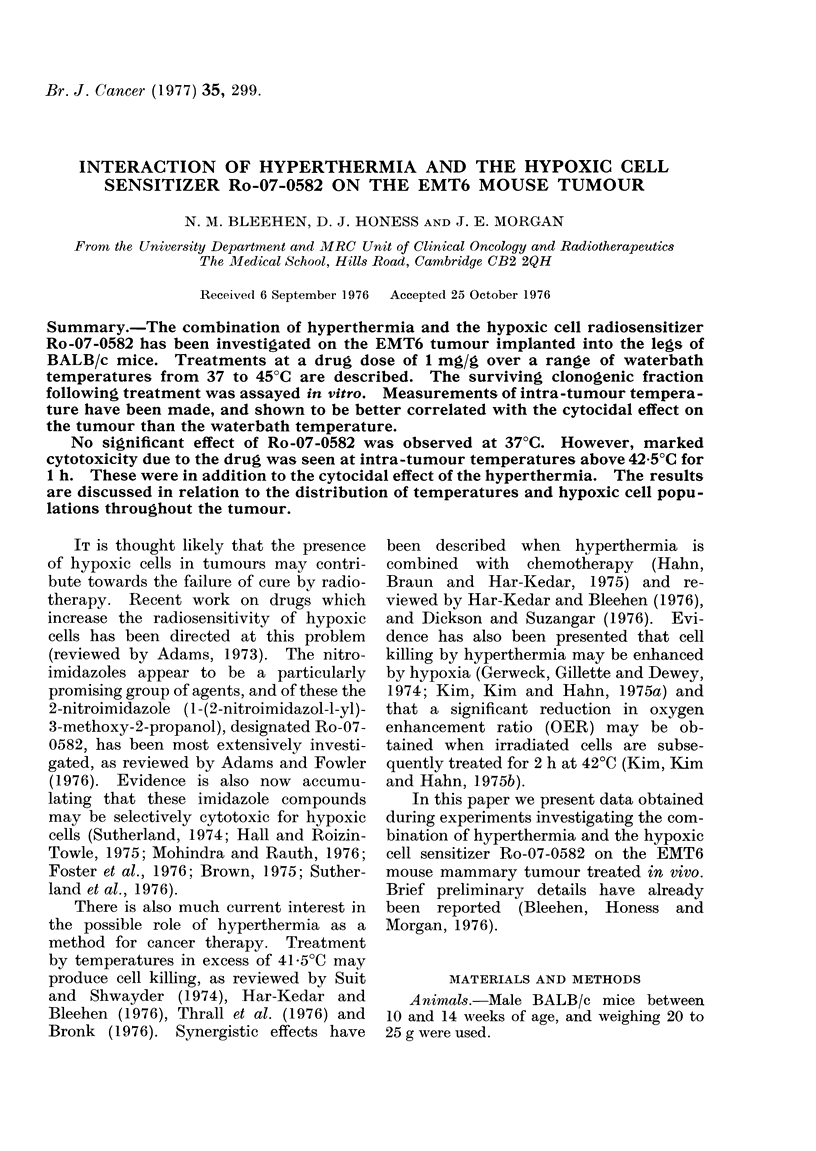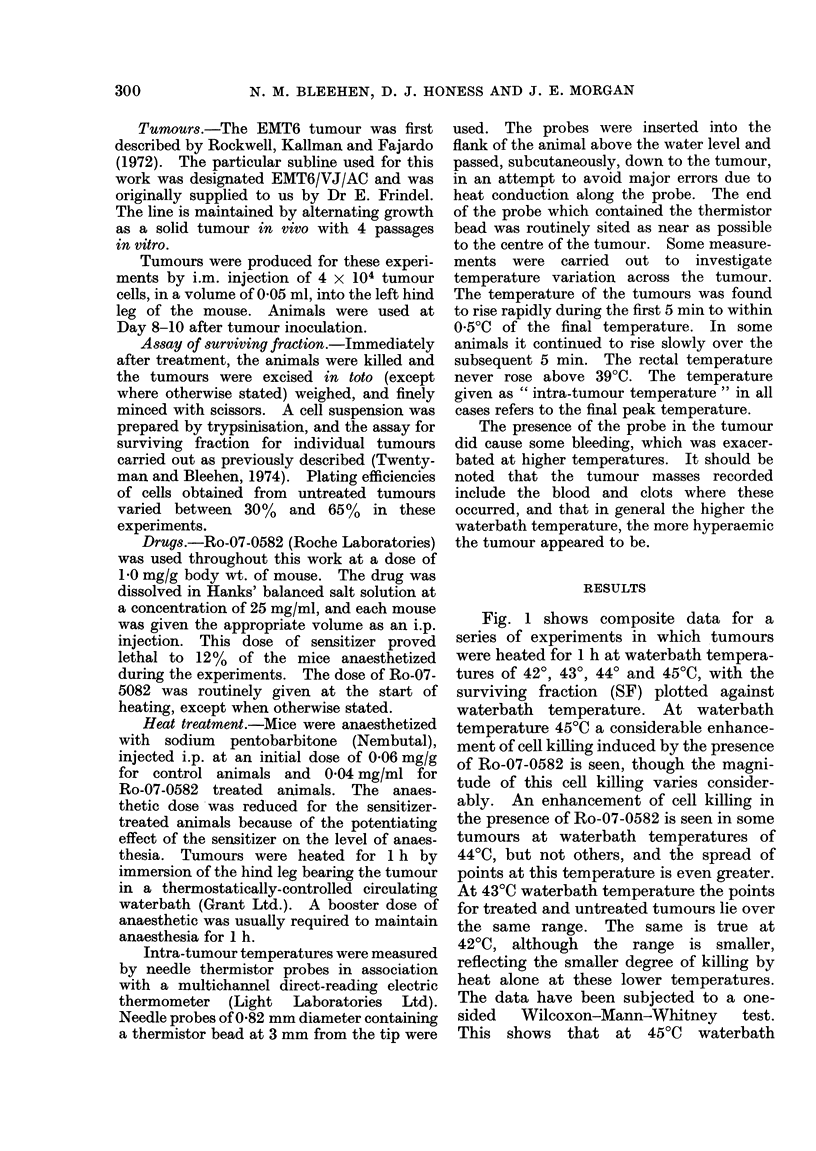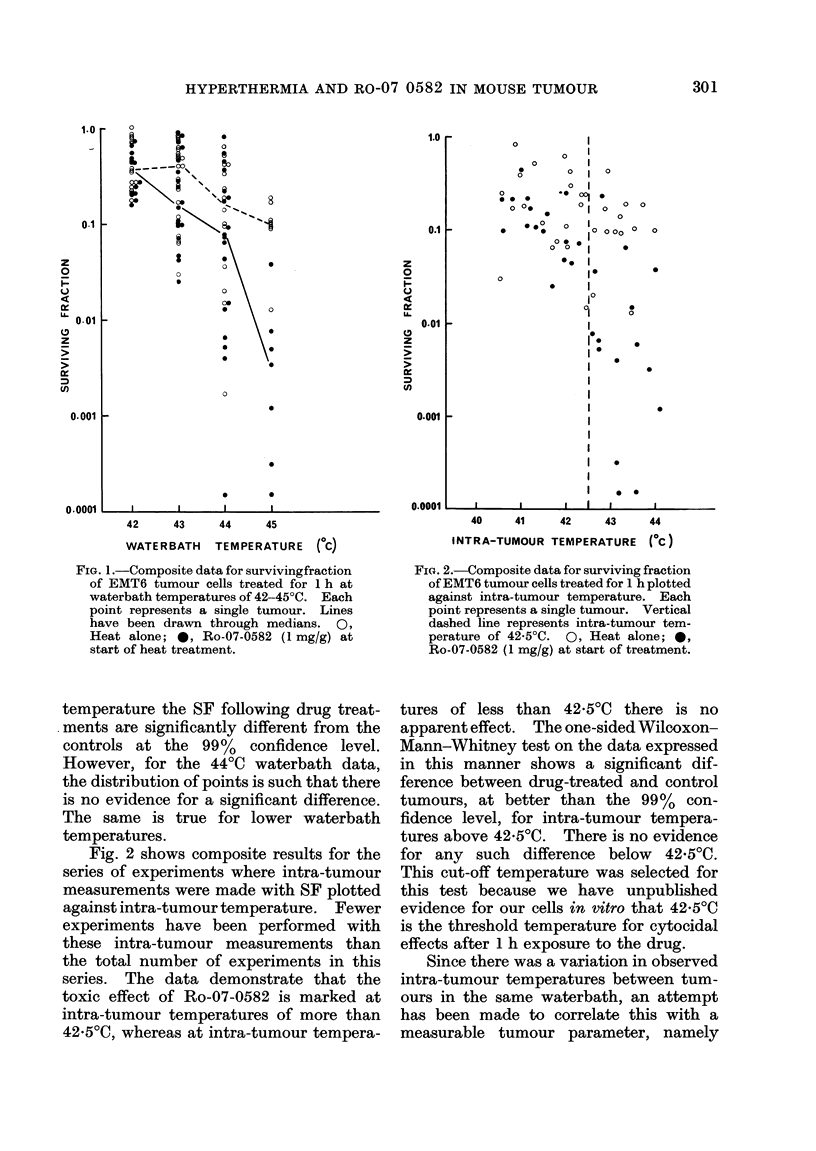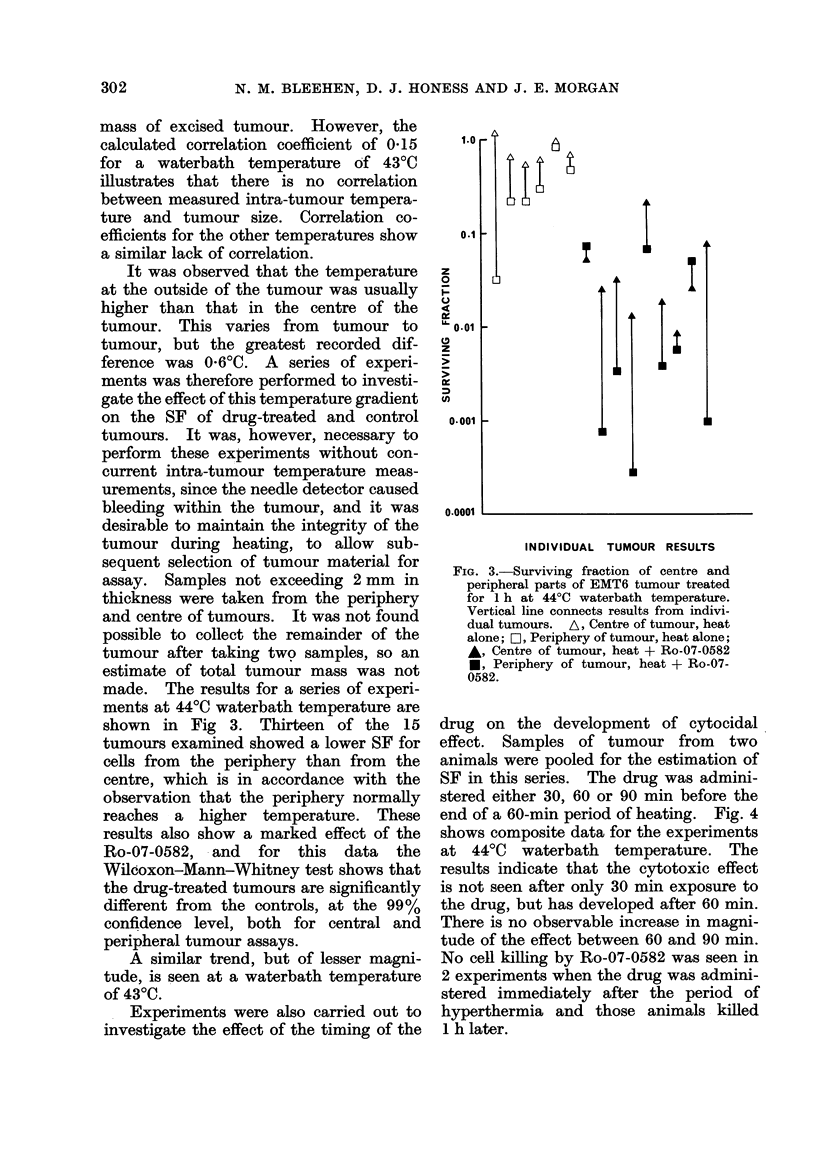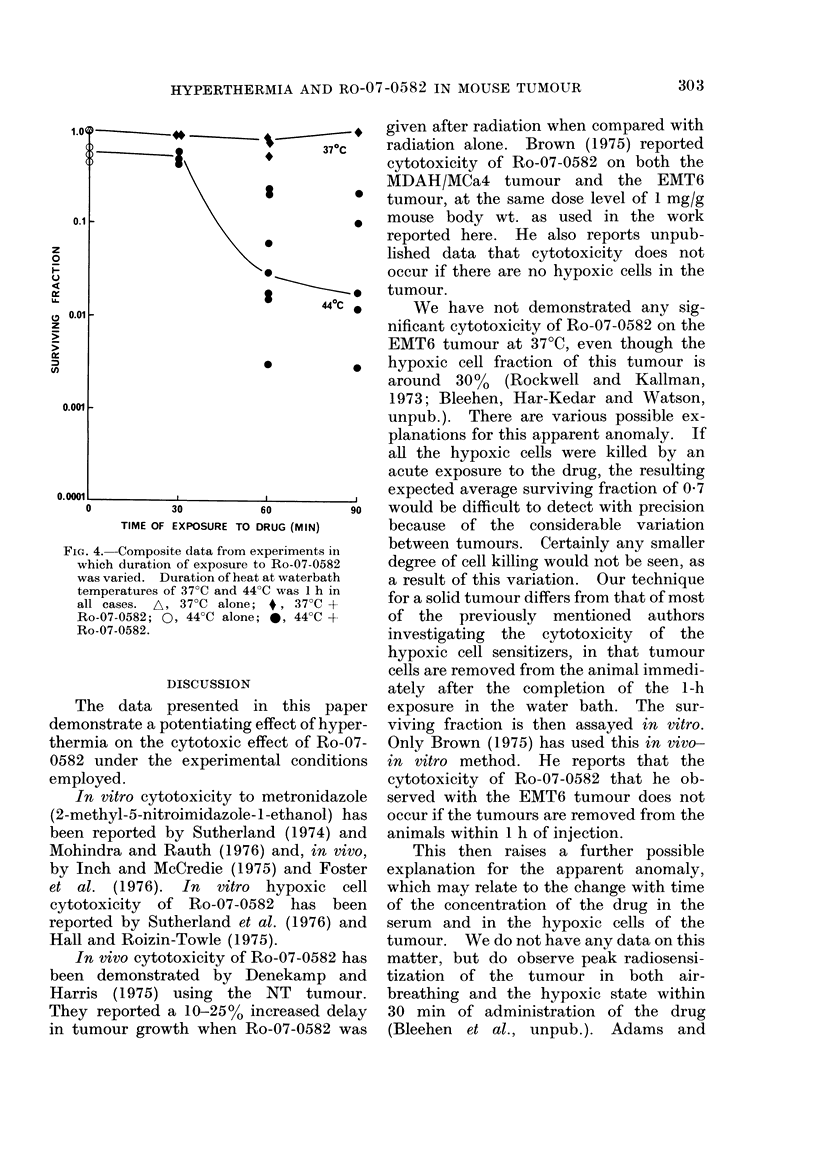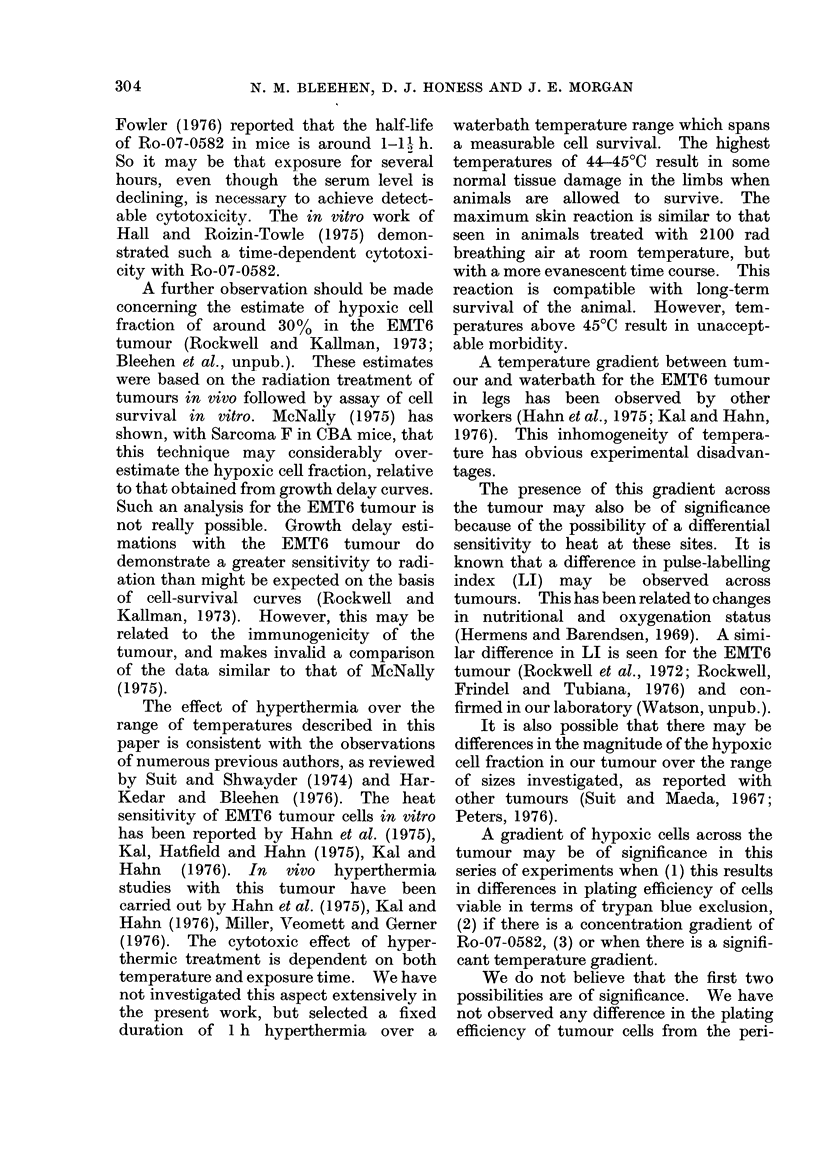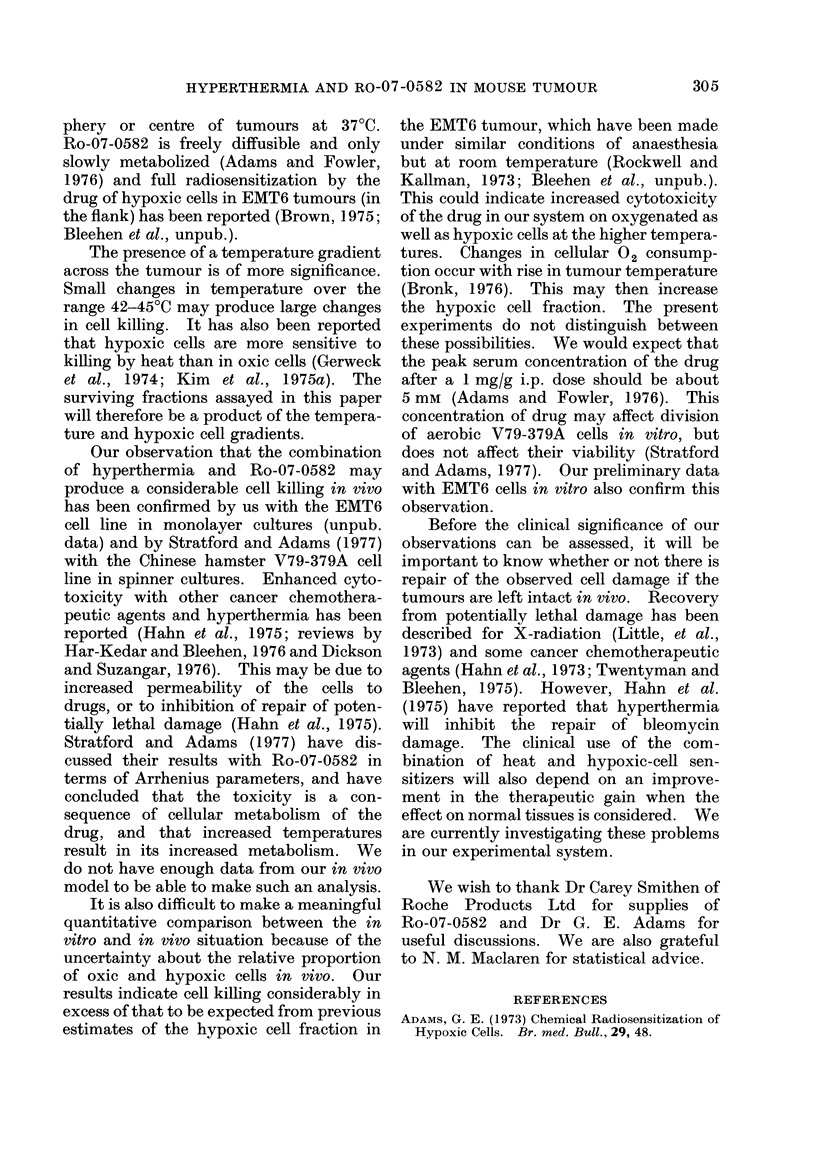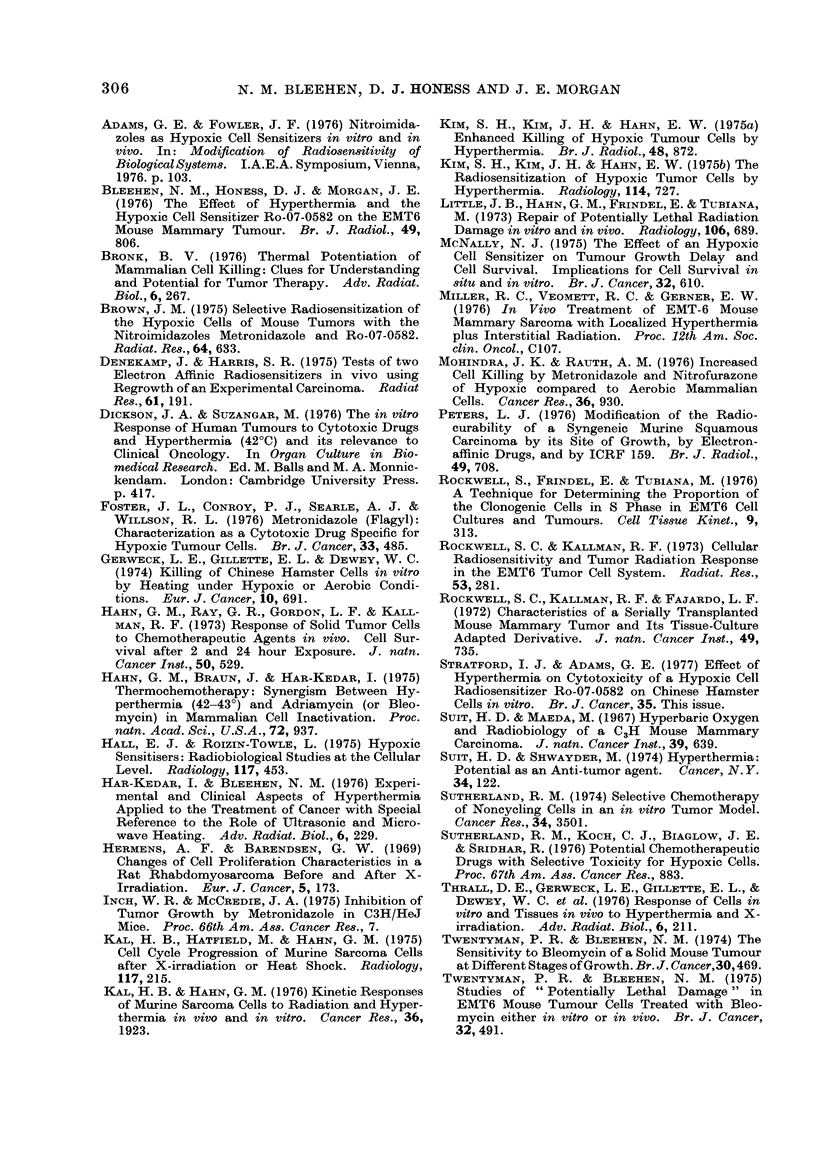# Interaction of hyperthermia and the hypoxic cell sensitizer Ro-07-0582 on the EMT6 mouse tumour.

**DOI:** 10.1038/bjc.1977.43

**Published:** 1977-03

**Authors:** N. M. Bleehen, D. J. Honess, J. E. Morgan

## Abstract

The combination of hyperthermia and the hypoxic cell radiosensitizer Ro-07-0582 has been investigated on the EMT6 tumour implanted into the legs of BALB/c mice. Treatments at a drug dose of 1 mg/g over a range of waterbath temperatures from 37 to 45 degrees C are described. The surviving clonogenic fraction following treatment was assayed in vitro. Measurements of intra-tumour temperature have been made, and shown to be better correlated with the cytocidal effect on the tumour than the waterbath temperature. No significant effect of Ro-07-0582 was observed at 37 degrees C. However, marked cytotoxicity due to the drug was seen at intra-tumour temperatures above 42-5 degrees C for 1 h. These were in addition to the cytocidal effect of the hyperthermia. The results are discussed in relation to the distribution of temperatures and hypoxic cell populations throughout the tumour.


*Br. J. Cancer* (1977) **35**, 299.

### INTERACTION OF HYPERTHERMIA AND THE HYPOXIC CELL SENSITIZER Ro-07-0582 ON THE EMT6 MOUSE TUMOUR

N. M. BLEEHEN, D. J. HONESS AND J. E. MORGAN

*From the University Department and MRC Unit of Clinical Oncology and Radiotherapeutics The Medical School, Hills Road, Cambridge CB2 2QH*

Received 6 September 1976 Accepted 25 October 1976

**Summary.—The combination of hyperthermia and the hypoxic cell radiosensitizer Ro-07-0582 has been investigated on the EMT6 tumour implanted into the legs of BALB/c mice. Treatments at a drug dose of 1 mg/g over a range of waterbath temperatures from 37 to 45°C are described. The surviving clonogenic fraction following treatment was assayed *in vitro*. Measurements of intra-tumour temperature have been made, and shown to be better correlated with the cytocidal effect on the tumour than the waterbath temperature.**

**No significant effect of Ro-07-0582 was observed at 37°C. However, marked cytotoxicity due to the drug was seen at intra-tumour temperatures above 42·5°C for 1 h. These were in addition to the cytocidal effect of the hyperthermia. The results are discussed in relation to the distribution of temperatures and hypoxic cell populations throughout the tumour.**

It is thought likely that the presence of hypoxic cells in tumours may contribute towards the failure of cure by radiotherapy. Recent work on drugs which increase the radiosensitivity of hypoxic cells has been directed at this problem (reviewed by Adams, 1973). The nitroimidazoles appear to be a particularly promising group of agents, and of these the 2-nitroimidazole (1-(2-nitroimidazol-l-yl)-3-methoxy-2-propanol), designated Ro-07-0582, has been most extensively investigated, as reviewed by Adams and Fowler (1976). Evidence is also now accumulating that these imidazole compounds may be selectively cytotoxic for hypoxic cells (Sutherland, 1974; Hall and Roizin-Towle, 1975; Mohindra and Rauth, 1976; Foster *et al.*, 1976; Brown, 1975; Sutherland *et al.*, 1976).

There is also much current interest in the possible role of hyperthermia as a method for cancer therapy. Treatment by temperatures in excess of 41·5°C may produce cell killing, as reviewed by Suit and Shwayder (1974), Har-Kedar and Bleehen (1976), Thrall *et al.* (1976) and Bronk (1976). Synergistic effects have been described when hyperthermia is combined with chemotherapy (Hahn, Braun and Har-Kedar, 1975) and reviewed by Har-Kedar and Bleehen (1976), and Dickson and Suzangar (1976). Evidence has also been presented that cell killing by hyperthermia may be enhanced by hypoxia (Gerweck, Gillette and Dewey, 1974; Kim, Kim and Hahn, 1975*a*) and that a significant reduction in oxygen enhancement ratio (OER) may be obtained when irradiated cells are subsequently treated for 2 h at 42°C (Kim, Kim and Hahn, 1975*b*).

In this paper we present data obtained during experiments investigating the combination of hyperthermia and the hypoxic cell sensitizer Ro-07-0582 on the EMT6 mouse mammary tumour treated *in vivo*. Brief preliminary details have already been reported (Bleehen, Honess and Morgan, 1976).

### MATERIALS AND METHODS

*Animals.*—Male BALB/c mice between 10 and 14 weeks of age, and weighing 20 to 25 g were used.

*Tumours.*—The EMT6 tumour was first described by Rockwell, Kallman and Fajardo (1972). The particular subline used for this work was designated EMT6/VJ/AC and was originally supplied to us by Dr E. Frindel. The line is maintained by alternating growth as a solid tumour *in vivo* with 4 passages *in vitro*.

Tumours were produced for these experiments by i.m. injection of $4 \times 10^4$ tumour cells, in a volume of 0·05 ml, into the left hind leg of the mouse. Animals were used at Day 8–10 after tumour inoculation.

*Assay of surviving fraction.*—Immediately after treatment, the animals were killed and the tumours were excised *in toto* (except where otherwise stated) weighed, and finely minced with scissors. A cell suspension was prepared by trypsinisation, and the assay for surviving fraction for individual tumours carried out as previously described (Twentyman and Bleehen, 1974). Plating efficiencies of cells obtained from untreated tumours varied between 30% and 65% in these experiments.

*Drugs.*—Ro-07-0582 (Roche Laboratories) was used throughout this work at a dose of 1·0 mg/g body wt. of mouse. The drug was dissolved in Hanks' balanced salt solution at a concentration of 25 mg/ml, and each mouse was given the appropriate volume as an i.p. injection. This dose of sensitizer proved lethal to 12% of the mice anaesthetized during the experiments. The dose of Ro-07-5082 was routinely given at the start of heating, except when otherwise stated.

*Heat treatment.*—Mice were anaesthetized with sodium pentobarbitone (Nembutal), injected i.p. at an initial dose of 0·06 mg/g for control animals and 0·04 mg/ml for Ro-07-0582 treated animals. The anaesthetic dose was reduced for the sensitizer-treated animals because of the potentiating effect of the sensitizer on the level of anaesthesia. Tumours were heated for 1 h by immersion of the hind leg bearing the tumour in a thermostatically-controlled circulating waterbath (Grant Ltd.). A booster dose of anaesthetic was usually required to maintain anaesthesia for 1 h.

Intra-tumour temperatures were measured by needle thermistor probes in association with a multichannel direct-reading electric thermometer (Light Laboratories Ltd). Needle probes of 0·82 mm diameter containing a thermistor bead at 3 mm from the tip were used. The probes were inserted into the flank of the animal above the water level and passed, subcutaneously, down to the tumour, in an attempt to avoid major errors due to heat conduction along the probe. The end of the probe which contained the thermistor bead was routinely sited as near as possible to the centre of the tumour. Some measurements were carried out to investigate temperature variation across the tumour. The temperature of the tumours was found to rise rapidly during the first 5 min to within 0·5°C of the final temperature. In some animals it continued to rise slowly over the subsequent 5 min. The rectal temperature never rose above 39°C. The temperature given as " intra-tumour temperature " in all cases refers to the final peak temperature.

The presence of the probe in the tumour did cause some bleeding, which was exacerbated at higher temperatures. It should be noted that the tumour masses recorded include the blood and clots where these occurred, and that in general the higher the waterbath temperature, the more hyperaemic the tumour appeared to be.

### RESULTS

Fig. 1 shows composite data for a series of experiments in which tumours were heated for 1 h at waterbath temperatures of 42°, 43°, 44° and 45°C, with the surviving fraction (SF) plotted against waterbath temperature. At waterbath temperature 45°C a considerable enhancement of cell killing induced by the presence of Ro-07-0582 is seen, though the magnitude of this cell killing varies considerably. An enhancement of cell killing in the presence of Ro-07-0582 is seen in some tumours at waterbath temperatures of 44°C, but not others, and the spread of points at this temperature is even greater. At 43°C waterbath temperature the points for treated and untreated tumours lie over the same range. The same is true at 42°C, although the range is smaller, reflecting the smaller degree of killing by heat alone at these lower temperatures. The data have been subjected to a one-sided Wilcoxon–Mann–Whitney test. This shows that at 45°C waterbath

FIG. 1.—Composite data for surviving fraction of EMT6 tumour cells treated for 1 h at waterbath temperatures of 42–45°C. Each point represents a single tumour. Lines have been drawn through medians. ○, Heat alone; ●, Ro-07-0582 (1 mg/g) at start of heat treatment.

FIG. 2.—Composite data for surviving fraction of EMT6 tumour cells treated for 1 h plotted against intra-tumour temperature. Each point represents a single tumour. Vertical dashed line represents intra-tumour temperature of 42·5°C. ○, Heat alone; ●, Ro-07-0582 (1 mg/g) at start of treatment.

temperature the SF following drug treatments are significantly different from the controls at the 99% confidence level. However, for the 44°C waterbath data, the distribution of points is such that there is no evidence for a significant difference. The same is true for lower waterbath temperatures.

Fig. 2 shows composite results for the series of experiments where intra-tumour measurements were made with SF plotted against intra-tumour temperature. Fewer experiments have been performed with these intra-tumour measurements than the total number of experiments in this series. The data demonstrate that the toxic effect of Ro-07-0582 is marked at intra-tumour temperatures of more than 42·5°C, whereas at intra-tumour temperatures of less than 42·5°C there is no apparent effect. The one-sided Wilcoxon–Mann–Whitney test on the data expressed in this manner shows a significant difference between drug-treated and control tumours, at better than the 99% confidence level, for intra-tumour temperatures above 42·5°C. There is no evidence for any such difference below 42·5°C. This cut-off temperature was selected for this test because we have unpublished evidence for our cells *in vitro* that 42·5°C is the threshold temperature for cytocidal effects after 1 h exposure to the drug.

Since there was a variation in observed intra-tumour temperatures between tumours in the same waterbath, an attempt has been made to correlate this with a measurable tumour parameter, namely mass of excised tumour. However, the calculated correlation coefficient of 0·15 for a waterbath temperature of 43°C illustrates that there is no correlation between measured intra-tumour temperature and tumour size. Correlation coefficients for the other temperatures show a similar lack of correlation.

It was observed that the temperature at the outside of the tumour was usually higher than that in the centre of the tumour. This varies from tumour to tumour, but the greatest recorded difference was 0·6°C. A series of experiments was therefore performed to investigate the effect of this temperature gradient on the SF of drug-treated and control tumours. It was, however, necessary to perform these experiments without concurrent intra-tumour temperature measurements, since the needle detector caused bleeding within the tumour, and it was desirable to maintain the integrity of the tumour during heating, to allow subsequent selection of tumour material for assay. Samples not exceeding 2 mm in thickness were taken from the periphery and centre of tumours. It was not found possible to collect the remainder of the tumour after taking two samples, so an estimate of total tumour mass was not made. The results for a series of experiments at 44°C waterbath temperature are shown in Fig 3. Thirteen of the 15 tumours examined showed a lower SF for cells from the periphery than from the centre, which is in accordance with the observation that the periphery normally reaches a higher temperature. These results also show a marked effect of the Ro-07-0582, and for this data the Wilcoxon–Mann–Whitney test shows that the drug-treated tumours are significantly different from the controls, at the 99% confidence level, both for central and peripheral tumour assays.

A similar trend, but of lesser magnitude, is seen at a waterbath temperature of 43°C.

Experiments were also carried out to investigate the effect of the timing of the

FIG. 3.—Surviving fraction of centre and peripheral parts of EMT6 tumour treated for 1 h at 44°C waterbath temperature. Vertical line connects results from individual tumours. △, Centre of tumour, heat alone; □, Periphery of tumour, heat alone; ▲, Centre of tumour, heat + Ro-07-0582 ■, Periphery of tumour, heat + Ro-07-0582.

drug on the development of cytocidal effect. Samples of tumour from two animals were pooled for the estimation of SF in this series. The drug was administered either 30, 60 or 90 min before the end of a 60-min period of heating. Fig. 4 shows composite data for the experiments at 44°C waterbath temperature. The results indicate that the cytotoxic effect is not seen after only 30 min exposure to the drug, but has developed after 60 min. There is no observable increase in magnitude of the effect between 60 and 90 min. No cell killing by Ro-07-0582 was seen in 2 experiments when the drug was administered immediately after the period of hyperthermia and those animals killed 1 h later.

FIG. 4.—Composite data from experiments in which duration of exposure to Ro-07-0582 was varied. Duration of heat at waterbath temperatures of 37°C and 44°C was 1 h in all cases. △, 37°C alone; ♦, 37°C + Ro-07-0582; ○, 44°C alone; ●, 44°C + Ro-07-0582.

### DISCUSSION

The data presented in this paper demonstrate a potentiating effect of hyperthermia on the cytotoxic effect of Ro-07-0582 under the experimental conditions employed.

*In vitro* cytotoxicity to metronidazole (2-methyl-5-nitroimidazole-1-ethanol) has been reported by Sutherland (1974) and Mohindra and Rauth (1976) and, *in vivo*, by Inch and McCredie (1975) and Foster *et al.* (1976). *In vitro* hypoxic cell cytotoxicity of Ro-07-0582 has been reported by Sutherland *et al.* (1976) and Hall and Roizin-Towle (1975).

*In vivo* cytotoxicity of Ro-07-0582 has been demonstrated by Denekamp and Harris (1975) using the NT tumour. They reported a 10–25% increased delay in tumour growth when Ro-07-0582 was given after radiation when compared with radiation alone. Brown (1975) reported cytotoxicity of Ro-07-0582 on both the MDAH/MCa4 tumour and the EMT6 tumour, at the same dose level of 1 mg/g mouse body wt. as used in the work reported here. He also reports unpublished data that cytotoxicity does not occur if there are no hypoxic cells in the tumour.

We have not demonstrated any significant cytotoxicity of Ro-07-0582 on the EMT6 tumour at 37°C, even though the hypoxic cell fraction of this tumour is around 30% (Rockwell and Kallman, 1973; Bleehen, Har-Kedar and Watson, unpub.). There are various possible explanations for this apparent anomaly. If all the hypoxic cells were killed by an acute exposure to the drug, the resulting expected average surviving fraction of 0·7 would be difficult to detect with precision because of the considerable variation between tumours. Certainly any smaller degree of cell killing would not be seen, as a result of this variation. Our technique for a solid tumour differs from that of most of the previously mentioned authors investigating the cytotoxicity of the hypoxic cell sensitizers, in that tumour cells are removed from the animal immediately after the completion of the 1-h exposure in the water bath. The surviving fraction is then assayed *in vitro*. Only Brown (1975) has used this *in vivo–in vitro* method. He reports that the cytotoxicity of Ro-07-0582 that he observed with the EMT6 tumour does not occur if the tumours are removed from the animals within 1 h of injection.

This then raises a further possible explanation for the apparent anomaly, which may relate to the change with time of the concentration of the drug in the serum and in the hypoxic cells of the tumour. We do not have any data on this matter, but do observe peak radiosensitization of the tumour in both air-breathing and the hypoxic state within 30 min of administration of the drug (Bleehen *et al.*, unpub.). Adams and

Fowler (1976) reported that the half-life of Ro-07-0582 in mice is around 1–1½ h. So it may be that exposure for several hours, even though the serum level is declining, is necessary to achieve detectable cytotoxicity. The *in vitro* work of Hall and Roizin-Towle (1975) demonstrated such a time-dependent cytotoxicity with Ro-07-0582.

A further observation should be made concerning the estimate of hypoxic cell fraction of around 30% in the EMT6 tumour (Rockwell and Kallman, 1973; Bleehen *et al.*, unpub.). These estimates were based on the radiation treatment of tumours *in vivo* followed by assay of cell survival *in vitro*. McNally (1975) has shown, with Sarcoma F in CBA mice, that this technique may considerably overestimate the hypoxic cell fraction, relative to that obtained from growth delay curves. Such an analysis for the EMT6 tumour is not really possible. Growth delay estimations with the EMT6 tumour do demonstrate a greater sensitivity to radiation than might be expected on the basis of cell-survival curves (Rockwell and Kallman, 1973). However, this may be related to the immunogenicity of the tumour, and makes invalid a comparison of the data similar to that of McNally (1975).

The effect of hyperthermia over the range of temperatures described in this paper is consistent with the observations of numerous previous authors, as reviewed by Suit and Shwayder (1974) and Har-Kedar and Bleehen (1976). The heat sensitivity of EMT6 tumour cells *in vitro* has been reported by Hahn *et al.* (1975), Kal, Hatfield and Hahn (1975), Kal and Hahn (1976). *In vivo* hyperthermia studies with this tumour have been carried out by Hahn *et al.* (1975), Kal and Hahn (1976), Miller, Veomett and Gerner (1976). The cytotoxic effect of hyperthermic treatment is dependent on both temperature and exposure time. We have not investigated this aspect extensively in the present work, but selected a fixed duration of 1 h hyperthermia over a waterbath temperature range which spans a measurable cell survival. The highest temperatures of 44–45°C result in some normal tissue damage in the limbs when animals are allowed to survive. The maximum skin reaction is similar to that seen in animals treated with 2100 rad breathing air at room temperature, but with a more evanescent time course. This reaction is compatible with long-term survival of the animal. However, temperatures above 45°C result in unacceptable morbidity.

A temperature gradient between tumour and waterbath for the EMT6 tumour in legs has been observed by other workers (Hahn *et al.*, 1975; Kal and Hahn, 1976). This inhomogeneity of temperature has obvious experimental disadvantages.

The presence of this gradient across the tumour may also be of significance because of the possibility of a differential sensitivity to heat at these sites. It is known that a difference in pulse-labelling index (LI) may be observed across tumours. This has been related to changes in nutritional and oxygenation status (Hermens and Barendsen, 1969). A similar difference in LI is seen for the EMT6 tumour (Rockwell *et al.*, 1972; Rockwell, Frindel and Tubiana, 1976) and confirmed in our laboratory (Watson, unpub.).

It is also possible that there may be differences in the magnitude of the hypoxic cell fraction in our tumour over the range of sizes investigated, as reported with other tumours (Suit and Maeda, 1967; Peters, 1976).

A gradient of hypoxic cells across the tumour may be of significance in this series of experiments when (1) this results in differences in plating efficiency of cells viable in terms of trypan blue exclusion, (2) if there is a concentration gradient of Ro-07-0582, (3) or when there is a significant temperature gradient.

We do not believe that the first two possibilities are of significance. We have not observed any difference in the plating efficiency of tumour cells from the periphery or centre of tumours at 37°C. Ro-07-0582 is freely diffusible and only slowly metabolized (Adams and Fowler, 1976) and full radiosensitization by the drug of hypoxic cells in EMT6 tumours (in the flank) has been reported (Brown, 1975; Bleehen *et al.*, unpub.).

The presence of a temperature gradient across the tumour is of more significance. Small changes in temperature over the range 42–45°C may produce large changes in cell killing. It has also been reported that hypoxic cells are more sensitive to killing by heat than in oxic cells (Gerweck *et al.*, 1974; Kim *et al.*, 1975*a*). The surviving fractions assayed in this paper will therefore be a product of the temperature and hypoxic cell gradients.

Our observation that the combination of hyperthermia and Ro-07-0582 may produce a considerable cell killing *in vivo* has been confirmed by us with the EMT6 cell line in monolayer cultures (unpub. data) and by Stratford and Adams (1977) with the Chinese hamster V79-379A cell line in spinner cultures. Enhanced cytotoxicity with other cancer chemotherapeutic agents and hyperthermia has been reported (Hahn *et al.*, 1975; reviews by Har-Kedar and Bleehen, 1976 and Dickson and Suzangar, 1976). This may be due to increased permeability of the cells to drugs, or to inhibition of repair of potentially lethal damage (Hahn *et al.*, 1975). Stratford and Adams (1977) have discussed their results with Ro-07-0582 in terms of Arrhenius parameters, and have concluded that the toxicity is a consequence of cellular metabolism of the drug, and that increased temperatures result in its increased metabolism. We do not have enough data from our *in vivo* model to be able to make such an analysis.

It is also difficult to make a meaningful quantitative comparison between the *in vitro* and *in vivo* situation because of the uncertainty about the relative proportion of oxic and hypoxic cells *in vivo*. Our results indicate cell killing considerably in excess of that to be expected from previous estimates of the hypoxic cell fraction in the EMT6 tumour, which have been made under similar conditions of anaesthesia but at room temperature (Rockwell and Kallman, 1973; Bleehen *et al.*, unpub.). This could indicate increased cytotoxicity of the drug in our system on oxygenated as well as hypoxic cells at the higher temperatures. Changes in cellular $O_2$ consumption occur with rise in tumour temperature (Bronk, 1976). This may then increase the hypoxic cell fraction. The present experiments do not distinguish between these possibilities. We would expect that the peak serum concentration of the drug after a 1 mg/g i.p. dose should be about 5 mM (Adams and Fowler, 1976). This concentration of drug may affect division of aerobic V79-379A cells *in vitro*, but does not affect their viability (Stratford and Adams, 1977). Our preliminary data with EMT6 cells *in vitro* also confirm this observation.

Before the clinical significance of our observations can be assessed, it will be important to know whether or not there is repair of the observed cell damage if the tumours are left intact *in vivo*. Recovery from potentially lethal damage has been described for X-radiation (Little, *et al.*, 1973) and some cancer chemotherapeutic agents (Hahn *et al.*, 1973; Twentyman and Bleehen, 1975). However, Hahn *et al.* (1975) have reported that hyperthermia will inhibit the repair of bleomycin damage. The clinical use of the combination of heat and hypoxic-cell sensitizers will also depend on an improvement in the therapeutic gain when the effect on normal tissues is considered. We are currently investigating these problems in our experimental system.

We wish to thank Dr Carey Smithen of Roche Products Ltd for supplies of Ro-07-0582 and Dr G. E. Adams for useful discussions. We are also grateful to N. M. Maclaren for statistical advice.

### REFERENCES

Adams, G. E. (1973) Chemical Radiosensitization of Hypoxic Cells. *Br. med. Bull.*, **29**, 48.

ADAMS, G. E. & FOWLER, J. F. (1976) Nitroimidazoles as Hypoxic Cell Sensitizers *in vitro* and *in vivo*. In: *Modification of Radiosensitivity of Biological Systems*. I.A.E.A. Symposium, Vienna, 1976. p. 103.

BLEEHEN, N. M., HONESS, D. J. & MORGAN, J. E. (1976) The Effect of Hyperthermia and the Hypoxic Cell Sensitizer Ro-07-0582 on the EMT6 Mouse Mammary Tumour. *Br. J. Radiol.*, **49**, 806.

BRONK, B. V. (1976) Thermal Potentiation of Mammalian Cell Killing: Clues for Understanding and Potential for Tumor Therapy. *Adv. Radiat. Biol.*, **6**, 267.

BROWN, J. M. (1975) Selective Radiosensitization of the Hypoxic Cells of Mouse Tumors with the Nitroimidazoles Metronidazole and Ro-07-0582. *Radiat. Res.*, **64**, 633.

DENEKAMP, J. & HARRIS, S. R. (1975) Tests of two Electron Affinic Radiosensitizers in vivo using Regrowth of an Experimental Carcinoma. *Radiat Res.*, **61**, 191.

DICKSON, J. A. & SUZANGAR, M. (1976) The *in vitro* Response of Human Tumours to Cytotoxic Drugs and Hyperthermia (42°C) and its relevance to Clinical Oncology. In *Organ Culture in Biomedical Research*. Ed. M. Balls and M. A. Monnickendam. London: Cambridge University Press. p. 417.

FOSTER, J. L., CONROY, P. J., SEARLE, A. J. & WILLSON, R. L. (1976) Metronidazole (Flagyl): Characterization as a Cytotoxic Drug Specific for Hypoxic Tumour Cells. *Br. J. Cancer*, **33**, 485.

GERWECK, L. E., GILLETTE, E. L. & DEWEY, W. C. (1974) Killing of Chinese Hamster Cells *in vitro* by Heating under Hypoxic or Aerobic Conditions. *Eur. J. Cancer*, **10**, 691.

HAHN, G. M., RAY, G. R., GORDON, L. F. & KALLMAN, R. F. (1973) Response of Solid Tumor Cells to Chemotherapeutic Agents *in vivo*. Cell Survival after 2 and 24 hour Exposure. *J. natn. Cancer Inst.*, **50**, 529.

HAHN, G. M., BRAUN, J. & HAR-KEDAR, I. (1975) Thermochemotherapy: Synergism Between Hyperthermia (42–43°) and Adriamycin (or Bleomycin) in Mammalian Cell Inactivation. *Proc. natn. Acad. Sci., U.S.A.*, **72**, 937.

HALL, E. J. & ROIZIN-TOWLE, L. (1975) Hypoxic Sensitisers: Radiobiological Studies at the Cellular Level. *Radiology*, **117**, 453.

HAR-KEDAR, I. & BLEEHEN, N. M. (1976) Experimental and Clinical Aspects of Hyperthermia Applied to the Treatment of Cancer with Special Reference to the Role of Ultrasonic and Microwave Heating. *Adv. Radiat. Biol.*, **6**, 229.

HERMENS, A. F. & BARENDSEN, G. W. (1969) Changes of Cell Proliferation Characteristics in a Rat Rhabdomyosarcoma Before and After X-Irradiation. *Eur. J. Cancer*, **5**, 173.

INCH, W. R. & MCCREDIE, J. A. (1975) Inhibition of Tumor Growth by Metronidazole in C3H/HeJ Mice. *Proc. 66th Am. Ass. Cancer Res.*, 7.

KAL, H. B., HATFIELD, M. & HAHN, G. M. (1975) Cell Cycle Progression of Murine Sarcoma Cells after X-irradiation or Heat Shock. *Radiology*, **117**, 215.

KAL, H. B. & HAHN, G. M. (1976) Kinetic Responses of Murine Sarcoma Cells to Radiation and Hyperthermia *in vivo* and *in vitro*. *Cancer Res.*, **36**, 1923.

KIM, S. H., KIM, J. H. & HAHN, E. W. (1975*a*) Enhanced Killing of Hypoxic Tumour Cells by Hyperthermia. *Br. J. Radiol.*, **48**, 872.

KIM, S. H., KIM, J. H. & HAHN, E. W. (1975*b*) The Radiosensitization of Hypoxic Tumor Cells by Hyperthermia. *Radiology*, **114**, 727.

LITTLE, J. B., HAHN, G. M., FRINDEL, E. & TUBIANA, M. (1973) Repair of Potentially Lethal Radiation Damage *in vitro* and *in vivo*. *Radiology*, **106**, 689.

MCNALLY, N. J. (1975) The Effect of an Hypoxic Cell Sensitizer on Tumour Growth Delay and Cell Survival. Implications for Cell Survival *in situ* and *in vitro*. *Br. J. Cancer*, **32**, 610.

MILLER, R. C., VEOMETT, R. C. & GERNER, E. W. (1976) *In Vivo* Treatment of EMT-6 Mouse Mammary Sarcoma with Localized Hyperthermia plus Interstitial Radiation. *Proc. 12th Am. Soc. clin. Oncol.*, C107.

MOHINDRA, J. K. & RAUTH, A. M. (1976) Increased Cell Killing by Metronidazole and Nitrofurazone of Hypoxic compared to Aerobic Mammalian Cells. *Cancer Res.*, **36**, 930.

PETERS, L. J. (1976) Modification of the Radiocurability of a Syngeneic Murine Squamous Carcinoma by its Site of Growth, by Electron-affinic Drugs, and by ICRF 159. *Br. J. Radiol.*, **49**, 708.

ROCKWELL, S., FRINDEL, E. & TUBIANA, M. (1976) A Technique for Determining the Proportion of the Clonogenic Cells in S Phase in EMT6 Cell Cultures and Tumours. *Cell Tissue Kinet.*, **9**, 313.

ROCKWELL, S. C. & KALLMAN, R. F. (1973) Cellular Radiosensitivity and Tumor Radiation Response in the EMT6 Tumor Cell System. *Radiat. Res.*, **53**, 281.

ROCKWELL, S. C., KALLMAN, R. F. & FAJARDO, L. F. (1972) Characteristics of a Serially Transplanted Mouse Mammary Tumor and Its Tissue-Culture Adapted Derivative. *J. natn. Cancer Inst.*, **49**, 735.

STRATFORD, I. J. & ADAMS, G. E. (1977) Effect of Hyperthermia on Cytotoxicity of a Hypoxic Cell Radiosensitizer Ro-07-0582 on Chinese Hamster Cells *in vitro*. *Br. J. Cancer*, **35**. This issue.

SUIT, H. D. & MAEDA, M. (1967) Hyperbaric Oxygen and Radiobiology of a $C_3H$ Mouse Mammary Carcinoma. *J. natn. Cancer Inst.*, **39**, 639.

SUIT, H. D. & SHWAYDER, M. (1974) Hyperthermia: Potential as an Anti-tumor agent. *Cancer, N.Y.* **34**, 122.

SUTHERLAND, R. M. (1974) Selective Chemotherapy of Noncycling Cells in an *in vitro* Tumor Model. *Cancer Res.*, **34**, 3501.

SUTHERLAND, R. M., KOCH, C. J., BIAGLOW, J. E. & SRIDHAR, R. (1976) Potential Chemotherapeutic Drugs with Selective Toxicity for Hypoxic Cells. *Proc. 67th Am. Ass. Cancer Res.*, 883.

THRALL, D. E., GERWECK, L. E., GILLETTE, E. L., & DEWEY, W. C. *et al.* (1976) Response of Cells *in vitro* and Tissues *in vivo* to Hyperthermia and X-irradiation. *Adv. Radiat. Biol.*, **6**, 211.

TWENTYMAN, P. R. & BLEEHEN, N. M. (1974) The Sensitivity to Bleomycin of a Solid Mouse Tumour at Different Stages of Growth. *Br. J. Cancer*, **30**, 469.

TWENTYMAN, P. R. & BLEEHEN, N. M. (1975) Studies of "Potentially Lethal Damage" in EMT6 Mouse Tumour Cells Treated with Bleomycin either *in vitro* or *in vivo*. *Br. J. Cancer*, **32**, 491.